# Full-laser-enabled clean hierarchical structuring and multifunctional synergy for high-performance in vivo 3D-printed implants

**DOI:** 10.1016/j.mtbio.2026.103095

**Published:** 2026-04-03

**Authors:** Qirui Zhang, Xinyue Zhang, Shanshan Liang, Jiaru Zhang, Qi Ma, Yiyang Wang, Xing Li, Fusong Yuan, Yingchun Guan, Huaming Wang

**Affiliations:** a School of Mechanical Engineering & Automation, Beihang University, Beijing, 102206, China; b Center of Digital Dentistry, Peking University Hospital of Stomatology, Beijing, 100081, China; c Center of Digital Dentistry, Department of Prosthodontics, Peking University School and Hospital of Stomatology, National Center for Stomatology, National Clinical Research Center for Oral Diseases, National Engineering Research Center of Oral Biomaterials and Digital Medical Devices, Beijing Key Laboratory of Digital Stomatology, NHC Key Laboratory of Digital Stomatology, Beijing, 100081, China; d School of Mechanical and Automotive Engineering, Ningbo University of Technology, Ningbo, 315336, China; e Hefei Innovation Research Institute of Beihang University, Anhui, 230012, China; f National Engineering Laboratory of Additive Manufacturing for Large Metallic Components, Beihang University, 37 Xueyuan Road, Beijing, 100191, China

**Keywords:** 3D-printed implant, In vivo, Hierarchical structures, Multifunctional surfaces, Hybrid laser processing

## Abstract

The long-term success of dental implants is often compromised by bacterial infection and inadequate osseointegration. Conventional surface modifications typically address these issues separately, lacking synergy and long-term stability. Here, we present a full-laser-enabled strategy that combines laser polishing technology and femtosecond laser-induced periodic surface structures (LIPSS), followed by spatially guided silver nanoparticle (AgNP) deposition on 3D-printed titanium. The novelty of current integrated process mainly lies in hierarchical topography regulation and programmable antibacterial ion delivery in a single clean platform. The hierarchical structures guide fibroblast and osteoblast alignment, enhancing cell adhesion and osteogenic differentiation, while also mechanically stretching bacterial membranes to facilitate Ag^+^ entry. Critically, the hierarchical geometry modulates Ag^+^ release kinetics, preventing burst release and enabling sustained antibacterial action. In vitro experiments have showed a 74.4% reduction in *Porphyromonas gingivalis* biofilm formation, a 300% increase in effective Ag^+^ release duration for ensuring sustained antibacterial efficacy and a 37.6% increase in gingival fibroblast proliferation, while in vivo animal experiments were conducted using Beagle dogs and have confirmed both the reduction of peri-implant inflammation and the enhancement of osseointegration as evidenced by a 38.7% increase in bone-implant contact ratio. This pioneering work unveils a scalable and synergistically optimized methodology for additive manufacturing of next-generation bioactive implants, enabling patient-specific customization of biomechanics and bioactivity.

## Graphical abstract

**Figure:**
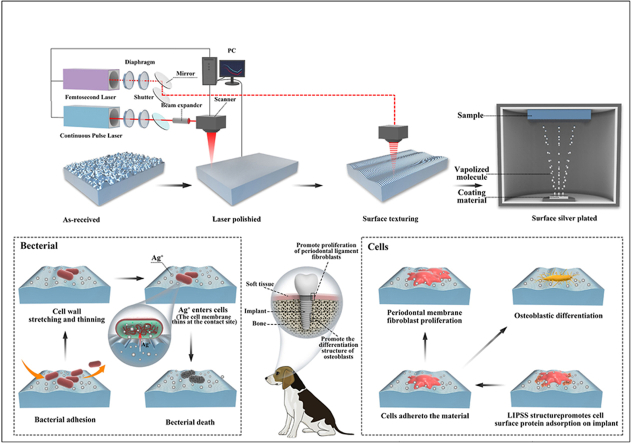

## 1 Introduction

The long-term clinical success of dental implants hinges on their ability to simultaneously promote osseointegration and resist bacterial colonization. Additive manufacturing (AM) has emerged as a promising route to produce personalized implants due to its design flexibility and structural adaptability [[1], [2], [3]]. However, surface defects of AM implants—including residual powder particles, irregular topography, and heterogeneous microstructures—will damage both soft and hard tissue integration, while also increasing the risk of bacterial adhesion and peri-implantitis [[4], [5], [6], [7]].

To enhance implant-tissue interactions, researchers have proposed various surface engineering strategies such as bioactive coatings [[8], [9], [10], [11]] and micro/nanostructuring to modulate cell adhesion, osteogenic differentiation, and immune responses [[12], [13], [14]]. In particular, topographical cues like nanopillars and spikes have successfully demonstrated potential to physically disrupt bacterial membranes [[15], [16], [17]], while certain hierarchical structures have been shown to simultaneously promote osteogenic differentiation and reduce bacterial adhesion by altering cellular phenotypes [18,19]. Nonetheless, most of recent methods target either bioactivity or antibacterial performance independently, and typically rely on physically or chemically isolated mechanisms [14]. Chemical-based antibacterial approaches, such as antibiotic or silver ion coatings, often suffer from burst release, short-term efficacy, or cytotoxicity. Simultaneously, top-down nanofabrication techniques include electron beam and X-ray lithography offer limited scalability and clinical applicability due to high complexity and cost [20,21]. Furthermore, the synergistic interplay between surface topography and antibacterial agents, especially in terms of how micro/nanostructures regulate ion release and cellular behavior, remains insufficiently explored.

**Fig. 1:**
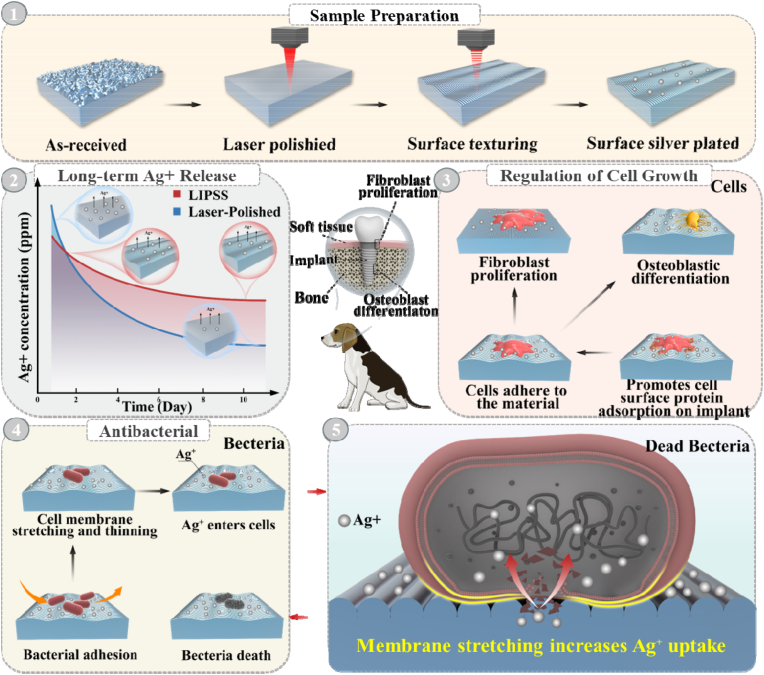
Schematic illustration of antimicrobial activity, and osseointegration fixation.

To address these limitations, we propose a hybrid laser-based surface engineering platform (Fig. 1, (1)), which sequentially integrates laser precision polishing of 3D-printed titanium dental implant, and femtosecond laser-induced periodic surface structures (LIPSS) with spatially guided Ag nanoparticle (AgNP) deposition. This revolutionary strategy enables simultaneous regulation of surface morphology, hierarchical micro/nanotopography, therapeutic functionalization, and silver nanoparticle embedding in a single clean scalable process, achieving both osseointegration and infection resistance (Fig. S1). As shown in Fig. 1 (2), the hierarchical surface regulates Ag^+^ release kinetics, mitigating burst release and maintaining long-term antibacterial activity below cytotoxic thresholds. In parallel, the LIPSS features direct fibroblast and osteoblast alignment (Fig. 1 (3)), while also deforming bacterial membranes and enhancing Ag^+^ permeability (Fig. 1 (4,5)). This integrated mechanism enables synergistic biological and antibacterial responses that surpass conventional single-function designs. By leveraging both structural and chemical cues in a programmable and scalable manner, our approach offers a clinically relevant pathway to multifunctional titanium implant surfaces.

## 2 Experimental section

### 2.1 Sample preparation

Ti6Al4V coupons were fabricated on a self-developed LPBF system. The laser power was 160 W, the scan speed was 1200 mm s^−1^, the hatch spacing was 100 μm, the laser spot diameter was 80 μm, and the nominal layer thickness was 30 μm.

This study utilized medical-grade titanium blocks (10 mm × 10 mm × 3 mm) and screw-shaped implants produced via laser additive manufacturing. A schematic of the precision motion platform employed for laser polishing and femtosecond micro/nanostructuring is presented in Fig. S1.

Laser polishing was performed using a nanosecond pulsed fiber laser (SPI red ENERGY G4, pulse duration 150 ns, wavelength 1064 nm, repetition frequency 300 kHz, overlap ratio 40%, spot size 50 μm) under argon protection, with an optimized scanning speed of 230 mm/s. A shadow scanning mode was applied, covering a 100 × 100 mm^2^ irradiation area.

Subsequently, micro/nanostructures were fabricated using a Yb-solid-state femtosecond laser (Pharos, Light Conversion) with 230 fs, 1030 nm pulses. The Gaussian beam diameter was approximately 35 μm, and processing was conducted at normal incidence in ambient air. After laser structuring, samples were ultrasonically cleaned in ethanol. A 10 nm silver film was then deposited using a high-vacuum metal coating system and converted into Ag nanoparticles by annealing at 200 °C for 1 h.

All processing steps were carried out on a self-developed five-axis laser system that integrates continuous-wave polishing and femtosecond patterning. This platform enables precise surface modification of both planar and complex implant geometries within a single workflow, ensuring scalability and clinical relevance.

### 2.2 Surface characterization

Surface morphology of the samples was observed using field-emission scanning electron microscopy (FE-SEM, Quanta 450 FEG, FEI, USA). Surface chemical composition was characterized by X-ray photoelectron spectroscopy (XPS, Thermo ESCALAB 250XI). Surface wettability was measured under ambient conditions using an OCA15EC system (DataPhysics, Germany). The static water contact angle (WCA) was measured with an automated pipetting system, depositing 5 μL of Hanks’ Balanced Salt Solution (HBSS) on the sample surface at room temperature.

### 2.3 Ag ion release

The release of Ag ions from coated samples immersed in PBS was monitored. Samples were immersed in 6 mL of PBS at 37 °C for 1 day, removed, and re-immersed in fresh PBS. This process was repeated 17 times to establish the time-release curve of Ag ions. The amount of Ag ions released in PBS solution was analyzed by inductively coupled plasma mass spectrometry (ICP-MS, ThermoFisher). Because protein binding and chloride/sulfide complexation in saliva- or serum-like media typically reduce the activity of free Ag^+^, PBS measurements are interpreted as a conservative upper bound for free-ion activity.

### 2.4 Corrosion resistance testing

Corrosion resistance was measured using an electrochemical workstation (Princeton Versa STAT 3F) with a standard three-electrode setup to determine the breakdown potential (Epit) corresponding to stable pitting. All samples served as working electrodes in 3.5 wt% NaCl solution at 25 °C, with a platinum counter electrode and a saturated calomel reference electrode. Corrosion current density (Icorr, mA/cm^2^), as well as the extrapolated and corrosion potential (Ecorr, VSCE), were derived directly from potentiodynamic polarization curves via the Tafel extrapolation method.

### 2.5 Friction testing

A UMT-3 rotary friction tester (Center for Tribology, USA) was used at 24 °C with a normal load of 1 N, sliding frequency of 1 Hz, and sliding distance of 1 mm. HBSS was used as the lubricant. To ensure repeatability, each test was conducted in triplicate.

### 2.6 Microhardness testing

Laser-polished specimens were sectioned in cross-section by electrical-discharge machining, then ground with SiC papers up to #2000 to obtain a flat cross-section. The polished blocks were cleaned in ethanol and air-dried before testing. Vickers microhardness was measured with a FVM-800 microhardness tester (Vickers diamond indenter; Future-Tech, Japan). Indentations were made normal to the surface along line scans from the treated surface toward the interior to obtain hardness profiles across the processed zone. A constant test load and dwell time were used for all measurements; results are reported in HV. To avoid edge and interaction effects, the first indent was placed at a standoff greater than three times the indentation diagonal from the free surface, and the spacing between adjacent indents was at least three times the diagonal.

### 2.7 In vitro cell evaluation

#### 2.7.1 Cell culture

The mouse calvarial preosteoblast cell line MC3T3-E1 (ATCC CRL-2593, RRID:CVCL_5440) and the human gingival fibroblast cell line HGF-1 (ATCC CRL-2014, RRID:CVCL_3710) were used to evaluate the in vitro biocompatibility and osteogenic performance of the laser-treated titanium surfaces. Both cell lines were obtained from the American Type Culture Collection (ATCC) and are widely accepted as standard models for assessing osteoblast differentiation and soft tissue response, respectively, in implant-related studies. Regular testing confirmed that all cell cultures were free of mycoplasma contamination throughout the experiments. MC3T3-E1 cells were cultured in α-minimal essential medium (α-MEM) containing 10% fetal bovine serum (FBS), while HGF-1 cells were cultured in Dulbecco's modified Eagle's medium (DMEM) with 10% FBS. Cells were maintained in an incubator at 37 °C with 5% CO_2_ and 95% humidity.

The selection of these well-characterized cell lines ensures biologically relevant and reproducible outcomes. Their use supports the validity of the conclusions drawn regarding the cytocompatibility, and osteogenic potential of the engineered implant surfaces.

#### 2.7.2 Cell viability and morphology

Specimens were sterilized under UV light for 24 h. MC3T3-E1 cells were seeded at a density of 7 × 10^4^ by adding 70 μL of cell suspension to the sample surface in a 24-well plate. Cell viability and morphology were assessed at 2, 4, and 24 h post-culture. Cell viability was measured using the CCK-8 assay following the manufacturer's instructions, and optical density was detected at 450 nm using a microplate reader. The morphology of cells was observed by SEM. After rinsing seeded scaffolds in phosphate-buffered saline (PBS) three times, cells were fixed in 4% paraformaldehyde for 30 min, rewashed in PBS, dehydrated in graded ethanol, vacuum-dried, sputter-coated with gold, and observed by SEM.

#### 2.7.3 Western blot analysis

To investigate the effect of surface topography on focal adhesion signaling, the expression of FAK and phosphorylated FAK (p-FAK) in MC3T3-E1 cells cultured on different samples was analyzed by western blot. After the designated culture period, total cellular proteins were extracted using an appropriate lysis buffer containing protease and phosphatase inhibitors. Equal amounts of protein were separated by SDS-PAGE and transferred onto PVDF membranes. After blocking, the membranes were incubated with primary antibodies against FAK, p-FAK, and β-actin, followed by incubation with the corresponding secondary antibodies. Protein bands were visualized using a chemiluminescence detection system, and β-actin was used as the internal loading control.

#### 2.7.4 ALP staining

To evaluate osteogenic differentiation, MC3T3-E1 cells were cultured on different samples for 7 and 14 days. At each time point, the cells were washed with PBS, fixed with 4% paraformaldehyde, and subjected to alkaline phosphatase (ALP) staining according to the manufacturer's instructions. Representative images were recorded to compare the osteogenic activity of cells on different surface morphologies.

#### 2.7.5 Live/dead staining and cell viability evaluation

To evaluate the cytocompatibility of AgNPs-free and AgNPs-coated samples, cells were seeded onto the sterilized specimens and cultured for 1 and 3 days. At each time point, cell viability was quantitatively evaluated using a standard viability assay according to the manufacturer's instructions. In parallel, live/dead staining was performed to visualize cell survival on different surfaces. Briefly, the cultured samples were rinsed with PBS and stained using a live/dead cell staining kit. Fluorescence images were then acquired using a fluorescence microscope, where live cells were stained green and dead cells were stained red. Quantitative analysis was performed based on the corresponding viability measurements at 1 and 3 days.

### 2.8 In vitro antibacterial assay

#### 2.8.1 Bacterial culture

The pathogenic bacterial strain used in this study was *Porphyromonas gingivalis* W83. Microorganisms were stored at −80 °C, inoculated on 5% sheep blood agar plates supplemented with vitamin K_1_ and hemin, and incubated anaerobically at 37 °C for 72 h using an anaerobic gas production bag (AnaeroPack, Mitsubishi Gas Chemical, Japan). Bacterial colonies were then transferred to 10 mL of brain heart infusion (BHI; Becton Dickinson, USA) broth supplemented with hemin (10 mg/mL) and menadione (5 mg/mL), and cultured anaerobically at 37 °C. Upon reaching the logarithmic growth phase, bacterial suspensions were diluted in sterile BHI broth to a final concentration of 1 × 10^7^ colony-forming units (CFU)/mL for use in experiments.

#### 2.8.2 SEM observation

Samples were placed in sterile 24-well plates with 1 mL of bacterial suspension (10^7^ CFU/mL) added to each well, fully immersing the sample, and incubated anaerobically at 37 °C for 24 h. Samples with biofilm formation were gently rinsed three times with 1 mL of sterile 0.89% NaCl solution, fixed in 2.5% glutaraldehyde at room temperature for 2 h, rinsed with ultrapure water, and dehydrated in graded ethanol solutions (30%, 50%, 70%, 80%, 90%, 95%, and 100%) for 10 min each. The samples were then air-dried for at least 24 h at room temperature, sputter-coated with gold, and observed by SEM (SU8010, Hitachi, Japan). All experiments were independently repeated three times (n = 3), with each experiment utilizing three technical replicates for statistical analysis.

To further evaluate the antibiofilm performance of the samples under a more clinically relevant oral microbial condition, a multispecies oral biofilm model was established using *Streptococcus gordonii*, *Fusobacterium nucleatum*, and *Porphyromonas gingivalis*. These three species were selected to represent early colonization, interspecies bridging, and late pathogenic colonization during oral biofilm development. Each strain was cultured under its appropriate growth conditions until the logarithmic growth phase, harvested, and resuspended in a supplemented BHI-based medium suitable for mixed oral bacterial growth. The bacterial suspensions were then mixed at a ratio of 1:1:1 to obtain a final total concentration of approximately 1 × 10^7^ CFU/mL.

Sterilized samples were placed in sterile 24-well plates, and 1 mL of the mixed bacterial suspension was added to each well to fully immerse the samples. The plates were incubated under anaerobic conditions at 37 °C for 48 h to allow multispecies biofilm formation. After incubation, the samples were gently rinsed three times with sterile 0.89% NaCl solution to remove loosely attached bacteria. For colony recovery assays, bacteria on the sample surfaces were collected and plated for agar culture. For morphological observation, the biofilm-covered samples were fixed in 2.5% glutaraldehyde at room temperature for 2 h, dehydrated in graded ethanol solutions (30%, 50%, 70%, 80%, 90%, 95%, and 100%) for 10 min each, air-dried, sputter-coated with gold, and examined by SEM (SU8010, Hitachi, Japan). All experiments were independently repeated three times (n = 3).

#### 2.8.3 Crystal violet staining for biofilm quantification

Samples were incubated with 1 mL of bacterial suspension (10^7^ CFU/mL) at 37 °C under anaerobic conditions. Biofilm biomass and adhered bacteria on the samples were measured by crystal violet staining. After 24 h of incubation, the medium was removed, and biofilm-covered samples were gently rinsed three times with PBS. Each well was then filled with 1 mL of 0.1% crystal violet solution and allowed to stand at room temperature for 20 min. Excess stain was removed by gently washing each well twice with PBS, and 1 mL of 99% ethanol was added to each well and maintained at room temperature for 15 min. The solution containing eluted crystal violet was transferred to a new microplate to estimate total biomass. Experiments were performed in triplicate to ensure methodological and biological reproducibility (n = 3).

#### 2.8.4 Statistical analysis

For all bacterial and cell experiments, three independent samples were tested for each experimental group, with three technical replicates per sample. All data are expressed as mean ± standard deviation, with error bars representing standard deviation in all figures.

#### 2.8.5 Oral implant animal model

Four six-week-old male Beagle dogs were obtained from the Animal Research Center of Peking University School of Stomatology and housed under specific pathogen-free conditions. The protocol was approved by the Biomedical Ethics Committee of Peking University (No. LA2022319) and complied with institutional and NIH guidelines. Two groups were studied: a peri-implant inflammation model assessed at 1 and 3 weeks, and a bone-growth group assessed at 8 weeks. A split-mouth allocation was used where feasible, with randomized site assignment and contralateral balancing. The unit of analysis was the implant site, and the number of implants per animal and per group is reported in the Results.

Animals were anesthetized by intraperitoneal sodium pentobarbital, and the surgical field received local lidocaine infiltration prior to incision. Physiologic monitoring included pulse oximetry, respiratory rate, heart rate, and temperature, and thermal support was provided throughout anesthesia. After molar extraction, implants were placed immediately into prepared sockets with the transmucosal collar exposed to the oral environment. Perioperative care followed institutional procedures. Analgesia consisted of an opioid regimen according to veterinary guidance, with predefined rescue analgesia available if pain scores exceeded threshold values. Prophylaxis and supportive care were provided as indicated by the attending veterinarian.

In the peri-implant inflammation model, an incision was made above the implant and a nonresorbable 7-0 suture was ligated around the implant neck and gently pressed apically into the gingival sulcus, with one end left intraorally to promote plaque accumulation; routine oral cleaning was withheld for the designated period. In the bone-growth group, implants were allowed to integrate without deliberate bacterial challenge. Regions of interest were prespecified. For soft-tissue outcomes, the region encircled the transmucosal collar adjacent to the implant surface. For bone outcomes, bone-to-implant contact was measured along predefined thread ranges, and peri-implant bone area fraction was quantified within a defined peri-implant band between selected threads. Allocation was randomized at the implant-site level, and histological scoring and image analysis were performed by assessors masked to group assignment.

Welfare monitoring followed ARRIVE and ISO 10993-2-aligned checklists. Animals were observed at least twice daily during the first three postoperative days and at least once daily thereafter, with records of appetite, activity, body weight, wound condition, oral hygiene status, and any adverse events. Pain was assessed using a standardized composite scale with predefined triggers for intervention. Humane endpoints were prespecified and included sustained anorexia, rapid weight loss, unrelieved pain at maximum allowable analgesia, respiratory compromise, or severe infection; animals meeting endpoint criteria were evaluated by the veterinarian for early termination.

At scheduled endpoints, animals were deeply anesthetized and euthanized by intravenous sodium pentobarbital overdose in accordance with institutional and international guidelines. Death was confirmed by absence of corneal reflex, apnea, and cardiac arrest. Mandibular segments containing the implant sites were immediately retrieved for histology and micro-CT.

#### 2.8.6 Histological staining

##### 2.8.6.1 Toluidine blue staining

For toluidine blue staining, sections were stained with 1% toluidine blue, rinsed, dehydrated, and sealed with resin, allowing differentiation of bone components, where newly formed calcified bone appeared deep blue, mature bone light blue, osteoid faint light blue, and the mineralization front as purple granules.

##### 2.8.6.2 Double fluorescence staining

Double fluorescence staining was used to assess dynamic bone formation, with sequential fluorochrome labeling enabling visualization of mineralization fronts at different time points.

##### 2.8.6.3 Hematoxylin and eosin (H&E) staining

Hematoxylin and eosin (H&E) staining was performed on 4 mm-thick vertical sections of the implant, which were then examined under an optical microscope (Olympus Corp, Shinjuku, Japan) to evaluate overall tissue structure and cellular morphology.

### 2.9 Micro-CT

Micro-CT imaging was performed using the Inveon Multi Modality system (Siemens, Germany) to evaluate peri-implant bone microstructure. Scanning was conducted at 80 kV and 500 μA, with a spatial resolution of 9.41 μm and a 360° rotational scan. Image reconstruction was completed using the DSF2 algorithm in Cobra software (Siemens, Germany). Quantitative analysis of trabecular bone parameters was performed using Inveon Image Research Workplace (Siemens, Germany), with a CT threshold range of 2000–4000 HU, to assess osseointegration and peri-implant bone remodeling.

## 3 Results and discussion

In laser powder bed fusion, the as-built surface quality is governed by the volumetric energy density $E_{v}=P/\left(v\cdot h\cdot l\right)$ together with scan strategy, hatch spacing, spot size, and layer thickness. Low $E_{v}$ or large hatch spacing promotes lack-of-fusion defects, adhered particles, and stair-stepping, whereas excessively high $E_{v}$ favors balling and spatter; both situations increase Ra. Because laser polishing is a controlled remelting step, the polishing window must be matched to the initial surface: rougher, particle-laden surfaces require higher line energy and/or larger overlap or multi-pass polishing to ensure track continuity, whereas smoother as-built surfaces require lower line energy and reduced overlap to suppress secondary melt redistribution and capillary-driven reflow, which would otherwise round features and cause geometry loss.

**Fig. 2:**
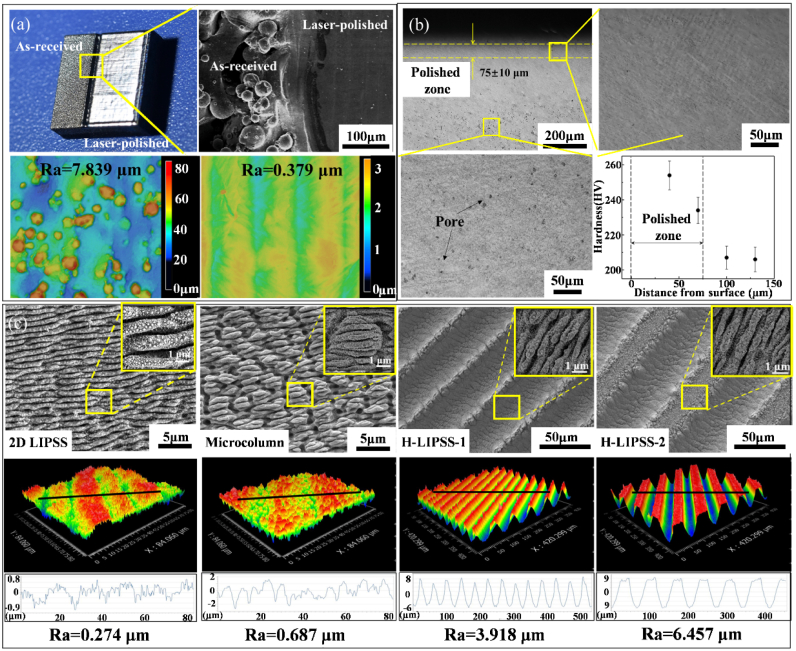
Surface properties, (a) Macro and micromorphology of sample surface before and after laser polishing; (b) Microstructure and microhardness distribution of cross-section before and after laser polishing; (c) 2D and 3D morphology of the four different micro/nano structures.

Consistent with this rationale, we examined the morphological evolution and near-surface refinement produced by laser polishing. As shown in Fig. 2(a), the as-received surface contained unmelted titanium particles with diameters of approximately 3–40 μm. After laser polishing, these particles were removed and a smooth, defect-free surface was obtained. The roughness $R_{a}$ decreased from 7.839 μm to 0.379 μm, reflecting effective remelting and material redistribution that minimized peak–valley height variations. Porosity in the polished layer was nearly eliminated, and a remelted zone of about 75±10 μm was formed. These structural changes correlate with the improved mechanical response on the same specimens, with Vickers microhardness increasing from 206 HV to 254 HV (Fig. 2(b)). The EBSD results (Fig. S2) show a shift of the grain-size distribution toward smaller equivalents, which supports a Hall–Petch-type strengthening of the processed zone.

The laser-polished surfaces were subsequently modified to generate four distinct types of AgNP-coated micro/nanostructures. Unless otherwise specified, all experiments were conducted on surfaces incorporating AgNP micro/nanostructures. As illustrated in Fig. 2(c), three primary micro/nanostructures were fabricated on the polished surface: laser-induced periodic surface structures (LIPSS), microcolumn, and hierarchical LIPSS structures (H-LIPSS-1 and H-LIPSS-2). These structures exhibited surface roughness values of 0.274 μm, 0.687 μm, 3.918 μm, and 6.457 μm, respectively. High-magnification imaging further revealed that AgNPs, with diameters ranging from 5 nm to 80 nm, were randomly distributed across the structured surfaces.

These findings underscore the precise control over surface morphology enabled by our hybrid approach. The variation in roughness levels and the spatial distribution of AgNPs across different micro/nanostructures provide a versatile platform for tailoring biological interactions, including cellular adhesion and antibacterial performance. This diverse structural design facilitates the optimization of both mechanical and biological functionalities within a single implant surface, potentially balancing smoothness for mechanical integrity with topographical modifications that enhance bioactivity and antimicrobial efficacy.

**Fig. 3:**
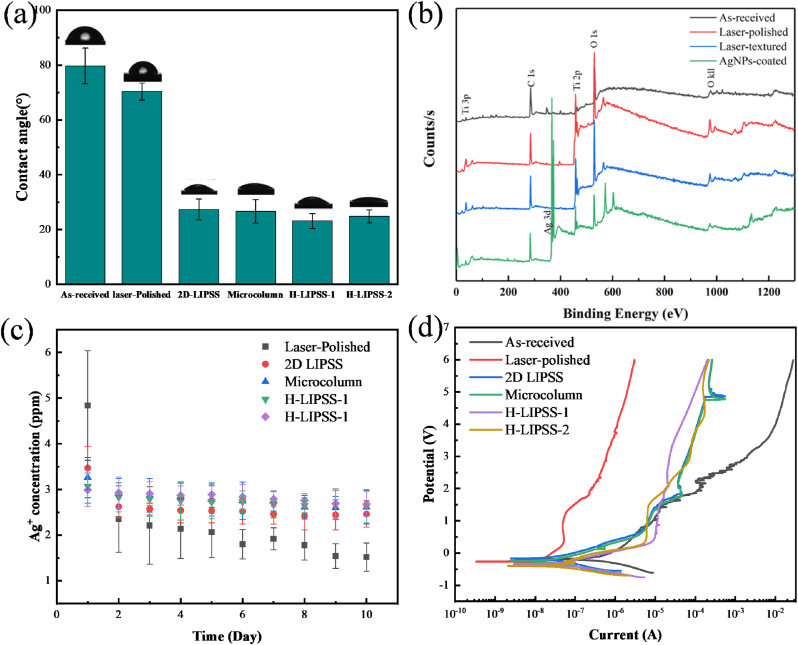
Comprehensive surface property characterization of different samples, (a) surface wettability; (b) surface chemical composition; (c) Ag ion release rate; and (d) electrochemical test results.

As shown in Fig. 3(a), the measured contact angles for the as-received surface, polished surface, and four distinct micro/nanostructures were 79.7°, 70.4°, 27.3°, 26.6°, 23.1°, and 24.8°, respectively. These results clearly demonstrate a significant enhancement in surface hydrophilicity after micro/nanostructuring, attributed to an increased effective contact area between the liquid and solid interface, in accordance with Wenzel's model [21,22]. X-ray photoelectron spectroscopy (XPS) results (Fig. 3(b)) indicated no significant alteration in surface chemistry resulting from laser polishing or microstructuring. However, following AgNP deposition, surface silver content increased markedly to 29.56%, verifying successful nanoparticle incorporation.

Fig. 3(c) presents the Ag-ion release profiles in PBS, showing an initial burst followed by a sustained-release stage. Such a release profile is beneficial for providing rapid antibacterial action at the early stage while maintaining prolonged ion availability over time, with concentrations remaining within literature-reported non-cytotoxic yet antibacterial-effective ranges [23]. Previous studies have further shown that micro/nanostructured surfaces can regulate local interfacial transport and thus extend the effective release duration of silver species [24]. The morphological basis for this behavior is supported by Fig. S3, which shows that after silver deposition and annealing, the initially continuous Ag coating transformed into discretely distributed AgNPs. These observations indicate that the engineered topographies provide a stable anchoring interface for AgNP immobilization, thereby enabling sustained ion release instead of rapid loss of loosely bound silver species.

The biosafety of the AgNP-coated surfaces was further evaluated by hemolysis assay and live/dead staining, as shown in Fig. S4. Although AgNP deposition led to a slight increase in the OD values associated with hemolysis compared with AgNPs-free samples, all groups remained below the 5% hemolysis threshold, indicating acceptable hemocompatibility. In addition, quantitative analysis of cell viability at 1 and 3 days, together with the representative live/dead fluorescence images, demonstrated that the AgNP-coated surfaces maintained good cytocompatibility, with the majority of cells remaining viable. These results suggest that the introduced AgNPs endow the surfaces with antibacterial functionality without causing an obvious loss of biosafety.

The corrosion resistance of modified surfaces was further investigated through electrochemical polarization tests (Fig. 3(d)). After laser polishing, the corrosion current density significantly decreased from 7.96 × 10^−7^ A/cm^2^ for the as-received surface to 1.32 × 10^−8^ A/cm^2^, representing a substantial 98.3% reduction. This improvement primarily resulted from the rapid solidification and elemental homogenization induced by laser processing, minimizing alloy element segregation and localized electrochemical activity, consistent with previous studies [4,25]. Subsequent microstructuring slightly increased surface roughness, thus moderately decreasing corrosion resistance. Notably, despite this slight decrease, the corrosion current density for all microstructured surfaces remained lower than 6.7% of the as-received sample, indicating a considerable overall enhancement in corrosion performance.

Tribological Behavior and Wear Mechanisms As illustrated in Fig. S5(a), the implant-bone interface undergoes radial micromotions under occlusal loading, underscoring the critical need for surface durability. Tribological performance was therefore evaluated (Fig. S5(b)). The coefficient of friction (COF) for the as-received sample increased stepwise, attributable to unmelted titanium particles disrupting direct surface contact. As these particles fractured under loading, surface contact increased, further raising the COF [23,25]. The laser-polished sample displayed an initial decrease in COF, followed by gradual elevation. Conversely, the micro/nanostructured surfaces consistently maintained stable COFs throughout testing. As shown in Fig. S5(c)), the COF values were 0.55 (as-received), 0.57 (polished), 0.46 (LIPSS), 0.47 (micropillar), 0.43 (H-LIPSS-1), and 0.41 (H-LIPSS-2), respectively. SEM analyses of worn surfaces (Fig. S5(d)) revealed prominent adhesive wear and abundant debris on the as-received surface. In contrast, polished and structured surfaces exhibited significantly less debris, indicating a shift towards abrasive wear. Importantly, hierarchical microstructures, particularly H-LIPSS-1 and H-LIPSS-2, effectively trapped wear debris within their grooves, mitigating third-body abrasion. Wear mechanism analyses (Fig. S5(e)) further clarified the tribological improvement of microstructured surfaces. These micro/nanostructures function as micro-reservoirs for lubricant retention, replenishing lubricant films when initial layers diminish or fail. By increasing lubricant contact area and reducing actual metal-to-metal contact, these structures significantly alleviate adhesive wear. Among tested configurations, H-LIPSS-2 demonstrated optimal debris-trapping capability due to its groove geometry, exhibiting the highest resistance to wear, and thus presenting a promising strategy for enhancing implant durability under physiological loading conditions.

**Fig. 4:**
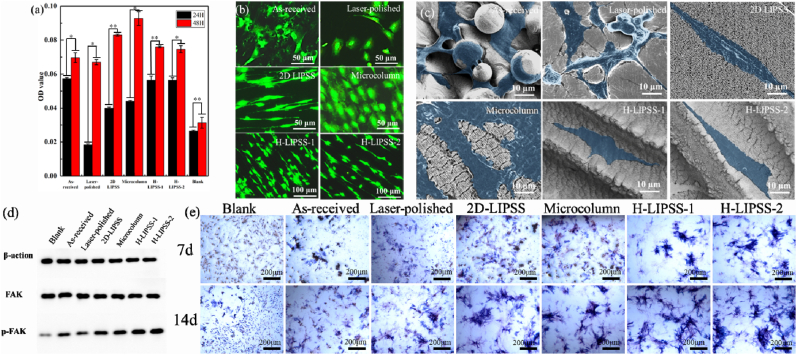
Effects of different surface morphology on osteoblasts, (a) The OD value of osteoblasts cultured for 24h and 48h; (b) Fluorescence images of osteoblasts cultured for 48h; (c) SEM images of osteoblasts cultured for 48h. (d) Western blot analysis of β-actin, FAK, and p-FAK expression in osteoblasts cultured on different surfaces; 4(e) ALP staining of osteoblasts cultured on different surfaces for 7 and 14 days. (*p-value <0.05, **p-value <0.01).

The LIPSS-modified surface plays a crucial role in modulating cellular behavior, particularly by enhancing osteogenic differentiation and promoting fibroblast adhesion. To investigate these effects, in vitro cell culture experiments were conducted using both osteoblasts and periodontal ligament fibroblasts, focusing on cell proliferation and morphology across six different surface conditions (Fig. 4) [26].

As shown in Fig. 4(a), the optical density (OD) values after 24 h and 48 h of osteoblast culture reveal that all laser-structured samples demonstrated enhanced proliferation compared to the as-received and polished controls. Notably, the micropillar (Microcolumn) surface exhibited the highest OD value at 48 h, indicating the most pronounced promotion of osteoblast proliferation. However, this increase in cell number was not accompanied by directional growth, suggesting a lack of topographical guidance. Fig. 4(b) presents fluorescence images of osteoblast morphology. Cells on the as-received and laser-polished surfaces showed randomly oriented, polygonal morphologies, which are typically indicative of weaker adhesion and less efficient differentiation. In contrast, cells cultured on the 2D LIPSS, H-LIPSS-1, and H-LIPSS-2 surfaces exhibited elongated morphologies aligned along the laser-induced patterns. This alignment reflects enhanced cytoskeletal organization and directional adhesion, which are key indicators of osteogenic potential [27]. While the micropillar surface supported extensive spreading, the cells retained a rounded or oval morphology, lacking orientation cues for differentiation. SEM observations in Fig. 4(c) further support these findings by revealing distinct cell-material interaction patterns. SEM images are shown with pseudocolor to make cell boundaries easier to see. The color has no quantitative meaning and does not change the underlying morphology. All measurements were performed on the original grayscale images; imaging parameters and scale bars are unchanged. On the as-received surface, unmelted titanium particles hindered effective cell spreading. The laser-polished surface, although smoother, exhibited only moderate filopodia extension. In contrast, the hierarchical LIPSS surfaces (H-LIPSS-1 and H-LIPSS-2) facilitated robust formation of lamellipodia and filopodia, with cells anchoring preferentially along the groove direction. These topographies provided spatial cues and physical anchoring sites, thereby improving adhesion and orientation. Among them, the H-LIPSS-2 surface displayed the most continuous and structured cell adhesion, suggesting that wider groove spacing allowed for enhanced pseudopodia attachment and cellular organization.

To further clarify whether the improved spreading and orientation of osteoblasts on the engineered surfaces were associated with enhanced adhesion signaling, the expression of FAK and phosphorylated FAK (p-FAK) was analyzed by western blot, as shown in Fig. 4(d). Compared with the as-received and laser-polished groups, the LIPSS structured surfaces exhibited increased FAK expression, while p-FAK showed a more pronounced topography-dependent enhancement, especially on the H-LIPSS-1 and H-LIPSS-2. This result indicates that the micro/nanostructured topographies, particularly the hierarchical LIPSS designs, more effectively activated focal adhesion signaling at the cell-material interface. Such enhanced p-FAK activation is consistent with the fluorescence and SEM observations, where osteoblasts on the hierarchical surfaces displayed more elongated morphologies, stronger alignment, and more extensive lamellipodia and filopodia formation.

The functional consequence of this enhanced adhesion response was further reflected in the ALP staining results (Fig. 4(e)). At both 7 and 14 days, the structured surfaces showed stronger ALP-positive staining than the as-received and laser-polished groups, with the hierarchical groups exhibiting the most intense and extensive staining. These findings suggest that the topography-induced enhancement in focal adhesion signaling was not limited to early cell attachment, but also promoted subsequent osteogenic differentiation. In this context, the hierarchical LIPSS surfaces appear to provide more effective spatial guidance and anchoring sites for osteoblasts, thereby establishing a more favorable osteogenic microenvironment. These results suggest that the topography-induced enhancement in osteoblast spreading and adhesion was further translated into improved osteogenic differentiation. Such behavior is consistent with the reported role of ordered micro/nanotopographies in promoting focal adhesion maturation, cytoskeletal organization, and osteogenic commitment [28,29].

Fig. S6(a) presents fibroblast proliferation rates on each sample after 24 and 48 h of culture. The optical density (OD) values progressively increased for all samples over time, indicating sustained cell growth. Notably, the four micro/nanostructured samples exhibited significantly enhanced proliferation compared to the AgNP-free and polished samples, demonstrating that these micro/nanostructures not only exhibit excellent biocompatibility but also ensure that surface-bound AgNPs do not induce cytotoxic effects. Among them, fibroblast proliferation was most pronounced on LIPSS-modified surfaces, with a 37.6% increase compared to the polished surface.

Fluorescence imaging of fibroblast morphology on different surfaces, as shown in Fig. S6(b), further illustrates the impact of surface topography. On micro/nanostructured surfaces, cells demonstrated highly aligned growth along the LIPSS grooves, indicating that the structured surfaces guided cell adhesion in a defined orientation. The LIPSS structures provided morphological cues that directed cell attachment and alignment, creating a more favorable environment for oriented fibroblast adhesion. This controlled cell organization is crucial for implant integration, as it may enhance soft-tissue adaptation to the implant surface while improving mechanical stability in vivo.

The SEM images in Fig. S6(c) further illustrate the distinct variations in cell morphology across different sample surfaces. On micro/nanostructured surfaces, cells exhibited extensive filopodia and lamellipodia, which primarily anchored to the LIPSS structures, demonstrating a significant enhancement in cell adhesion. Among these, cell attachment was most pronounced on the H-LIPSS-2 surface, where fibroblasts preferentially adhered to the micro-groove regions. Conversely, the H-LIPSS-1 surface exhibited a weaker influence on cell distribution, which may be attributed to differences in groove width—wider grooves offer more attachment space for cellular pseudopodia, facilitating better adhesion.

These findings align with previous studies indicating that micro/nanostructural cues significantly influence integrin-mediated cell anchoring, cytoskeletal organization, and downstream signaling pathways critical for osteogenic differentiation [30]. Furthermore, the hierarchical structures facilitated the initial adsorption of extracellular matrix proteins, creating a favorable microenvironment that promotes cell adhesion and proliferation [31]. Overall, the LIPSS-modified surfaces demonstrate significant potential in enhancing cellular behavior, contributing to improved implant integration and long-term stability.

Unlike conventional antibacterial strategies that rely solely on either chemical or physical mechanisms, the LIPSS-AgNPs approach establishes a robust and complementary antibacterial effect. The hierarchical micro/nanostructures significantly enhance bacterial membrane deformation and mechanical tension, leading to membrane stretching, disruption, and rupture, as confirmed by SEM observations. This mechanism aligns with previously reported bioinspired antimicrobial surfaces, such as nanopillar and nano-spike arrays, which physically compromise bacterial membrane integrity [32,33]. However, unlike traditional microstructures with limited antibacterial efficiency, the optimized hierarchical LIPSS configuration effectively amplifies bacterial membrane stresses, increasing bacterial susceptibility to mechanical disruption and facilitating Ag^+^ penetration, thereby maximizing bactericidal efficacy.

**Fig. 5:**
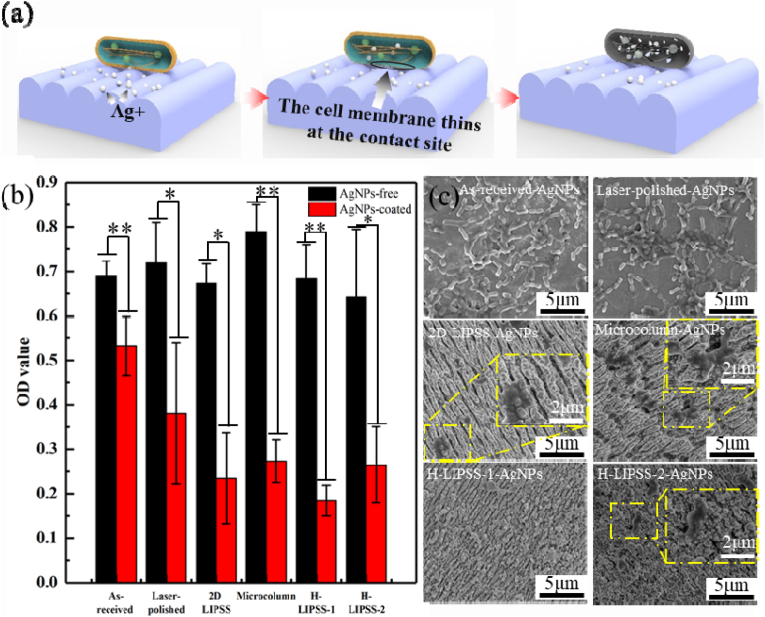
Antibacterial properties of different samples against P.gingivalis, (a) Schematic diagram of LIPSS structure for antibacterial activity; (b)The OD value of P.gingivalis cultured for 24h; (c) Bacterial morphology of P.gingivalis cultured for 24h. (*p-value <0.05, **p-value <0.01).

Additionally, the embedded AgNPs provide a sustained chemical antibacterial effect, effectively targeting residual viable bacteria that might otherwise withstand physical deformation. Our previous studies systematically optimized the Ag^+^ release kinetics [34,35], demonstrating that the hierarchical micro/nano-scale geometry of LIPSS enables controlled and prolonged Ag^+^ release, mitigating the rapid depletion and burst release effects commonly observed in conventional silver coatings. The nanostructures function as Ag^+^ reservoirs, ensuring a gradual and sustained antibacterial effect while minimizing cytotoxicity risks. As a result, this study achieves a bacterial inhibition rate of 74.4%, significantly surpassing the efficacy of either LIPSS or AgNPs alone. As shown in Fig. 5(a), The synergistic interplay between microstructural-induced bacterial membrane disruption and chemically mediated antibacterial activity constitutes the core innovation of this surface modification strategy, offering a dual-action approach for long-term infection resistance.

To assess the antibacterial performance of different surface modifications, Fig. 5(b) presents the antibacterial efficacy of the as-received, polished, and four micro/nanostructured samples without AgNPs against *Porphyromonas gingivalis* (*P. gingivalis*). The results demonstrate that surfaces without AgNPs exhibited minimal antibacterial effects, aligning with the well-established mechanism of silver nanoparticles in antibacterial activity. The bactericidal action of AgNPs primarily arises from the release of Ag ions, which carry a positive charge and interact electrostatically with the negatively charged bacterial cell membrane. This interaction promotes Ag ion adhesion to the bacterial cell wall or cytoplasmic membrane, increasing membrane permeability and ultimately compromising structural integrity. As a result, bacterial metabolism is inactivated, leading to cellular dysfunction or death. Following AgNP deposition, the antibacterial rates for the four micro/nanostructured surfaces increased significantly to 67.3%, 62%, 74.4%, and 63.1%, respectively, demonstrating a marked improvement in antibacterial efficacy compared to samples without AgNPs. This enhancement highlights the critical role of AgNP incorporation in improving antibacterial performance, making it a promising strategy for infection-resistant implant surfaces.

The observed variations in antibacterial efficacy suggest that surface morphology plays a crucial role in regulating both Ag ion release kinetics and bacterial adhesion behavior. Previous studies have shown that the LIPSS structure effectively inhibits *Escherichia coli* by trapping bacteria within its grooves, leading to membrane stretching, deformation, and eventual rupture [2,17]. However, in our experiments with *P. gingivalis*, the antibacterial effect of LIPSS alone appeared relatively weaker. This difference can be attributed to fundamental bacterial structural differences: unlike *E. coli*, which possesses an outer membrane that is more susceptible to physical disruption, *P. gingivalis* has a thick peptidoglycan layer in its cell wall. This feature grants *P. gingivalis* higher mechanical strength and rigidity, making it more resistant to direct structural damage induced by surface micro/nanostructures. Nevertheless, while the LIPSS grooves may not physically rupture the bacterial cell wall as effectively, they induce localized membrane stretching, facilitating Ag ion penetration through the mechanically weakened bacterial membrane. This enhances the bactericidal efficacy of AgNPs, demonstrating a synergistic antibacterial mechanism that combines physical and chemical effects. Notably, while the OD-based antibacterial rates were recorded as 67.3%, 62%, 74.4%, and 63.1%, visual inspection of bacterial morphology in Fig. 5(c) suggests that a significant portion of bacteria on microstructured surfaces exhibited membrane deformation, rupture, or collapse, appearing as flattened or fragmented cells. These non-viable bacteria were still included in the OD measurement, potentially underestimating the actual antibacterial effectiveness. This observation reinforces that micro/nanostructures not only possess inherent antibacterial properties but also amplify the effects of Ag ions by inducing bacterial membrane deformation, thereby significantly enhancing their bactericidal efficacy.

To further evaluate whether this antibacterial effect could be extended beyond single-species assays and better reflect the polymicrobial nature of the oral microenvironment, a multispecies oral biofilm model was additionally established using *Streptococcus gordonii*, *Fusobacterium nucleatum*, and *Porphyromonas gingivalis* under anaerobic conditions. As shown in Fig. S7, the structured surfaces, particularly the hierarchical micro/nanostructured groups, exhibited fewer recoverable colonies and markedly less compact biofilm accumulation after 48 h incubation compared with the control surface. SEM observations further revealed that bacteria on these engineered surfaces were more sparsely distributed and that the mixed-species biofilm architecture appeared more discontinuous and disorganized, in contrast to the denser and more coherent biofilm structures observed on the untreated surface. Moreover, confocal laser scanning microscopy (CLSM) provided complementary visualization of the spatial architecture of the formed biofilm, further supporting that the hierarchical surfaces suppressed biofilm continuity and reduced biofilm accumulation. These results indicate that the antibacterial effect of the hierarchical topographies is not restricted to a single periodontal pathogen, but can also be extended to a more clinically relevant mixed bacterial consortium. This improved antibiofilm behavior is likely related to the combined effects of reduced initial bacterial attachment, disturbance of interspecies bridging during biofilm development, and sustained Ag ion-mediated antibacterial protection. Therefore, the hierarchical AgNP-functionalized surfaces appear capable of suppressing multispecies oral biofilm formation under conditions that better mimic the in vivo oral microbial environment.

**Fig. 6:**
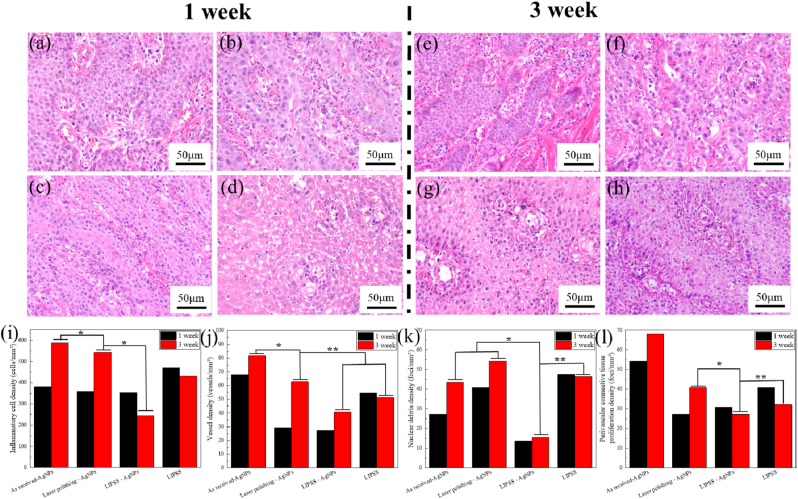
Results of H&E staining for implant groups, (a)(b)(c)(d) show outcomes at 1 week, (e)(f)(g)(h) show outcomes at 3 weeks. Among them, (a)(e) represent the original material - AgNPs; (b)(f) represent laser polishing - AgNPs; (c)(g) represent LIPSS - AgNPs; (d)(h) represent LIPSS; (i) inflammatory cell density; (j) vessel density; (k) nuclear debris density; and (l) perivascular connective tissue proliferation density.

As shown in Fig. 6, a Beagle root-defect model was established to compare in-vivo tissue responses across surface treatments. To create an infection-prone setting, implants were preconditioned to allow biofilm formation and a silk ligature was placed around the transmucosal collar at surgery. Early infection was assessed by microbiology at 1 and 3 weeks, and H&E sections within a prespecified peri-implant ROI confirmed leukocyte infiltration, vascular congestion/edema, and apical epithelial migration. A representative intraoral photograph illustrating edema, erythema, and exudate at week 1 is provided in Supplementary Fig. S8.

To confirm the peri-implant infection status, H&E staining was performed and histological responses were quantitatively analyzed in terms of inflammatory cell density, vessel density, nuclear debris density, and perivascular connective tissue proliferation density (Fig. 6). At 1 week, the LIPSS group showed the most pronounced inflammatory response, with high inflammatory cell density and elevated tissue damage-related indices, indicating that surface structuring alone was insufficient to prevent early bacterial challenge. In contrast, all AgNP-modified groups exhibited reduced inflammatory severity, confirming the anti-infective benefit of silver incorporation. Among them, the LIPSS + AgNPs group displayed the lowest inflammatory cell density and nuclear debris density, together with relatively mild perivascular connective tissue proliferation, suggesting the most favorable early tissue response.

At 3 weeks, the as-received + AgNPs and laser-polished + AgNPs groups still exhibited relatively high inflammatory and vascular indices, while the LIPSS group continued to show unfavorable histological changes. By comparison, the LIPSS + AgNPs group maintained the lowest overall inflammatory burden, indicating that the combination of LIPSS topography and AgNPs more effectively limited persistent bacterial stimulation and promoted improved peri-implant tissue healing over time.

Comparative analyses of different surface morphologies revealed that hierarchical micro/nano structures exert a more pronounced effect on osteoblast morphology and differentiation. The LIPSS-AgNPs surfaces significantly improved key bone regeneration indicators, including BV/TV, Tb.Th, and Tb.N in vivo, highlighting their superior osteogenic potential. Unlike conventional approaches, which typically emphasize either antibacterial or osteogenic properties, this hierarchical surface modification strategy successfully integrates both functionalities, offering a comprehensive and long-term solution for implant applications.

The clinical success of dental implants depends on their ability to simultaneously prevent bacterial infections and promote osseointegration. The LIPSS-AgNPs strategy presented in this study effectively addresses both challenges by leveraging topographical optimization and controlled AgNPs release. In vivo results from the Beagle dog tooth defect model further validate its efficacy, demonstrating superior infection resistance and enhanced bone integration compared to conventional implant surfaces.

**Fig. 7:**
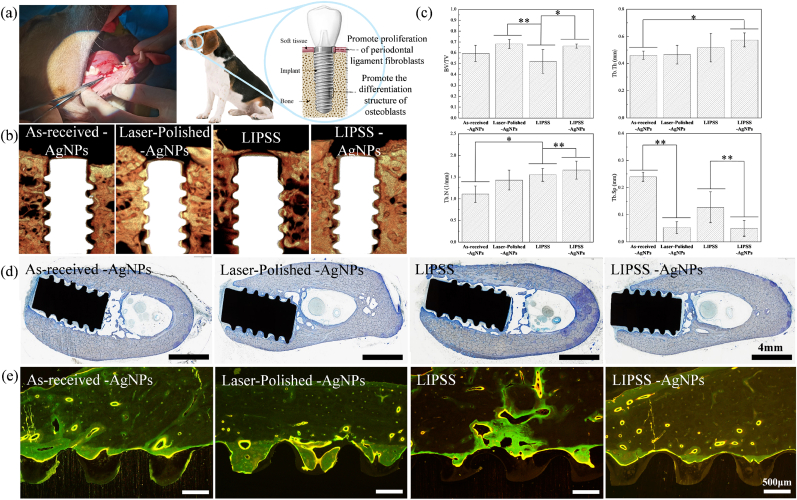
In vivo evaluation of implant osseointegration and bone regeneration in Beagle dogs, including (a) implantation in the oral cavity; (b) microscopic CT three-dimensional models; (c) corresponding bone tissue morphometry of various implants; (d) histological evaluation of hard tissue sections stained with toluidine blue to indicate in vivo bone integration; and (e) fluorescent labeling of newly formed bone around the implant, where green represents calcein labeling. (*p-value <0.05, **p-value <0.01).

In a separate experiment, we investigated bone integration. By the eighth week, microscopic CT (Fig. 7(b)) analysis revealed that implants with laser-modified surfaces exhibited significantly higher levels of regenerated bone compared to other groups, indicating superior in vivo osseointegration and osteogenic fixation capabilities. Quantitative analysis of new bone formation (Fig. 7(c)) further demonstrated that the LIPSS-AgNPs structure yielded the highest values in parameters such as BV/TV, Tb.Th, Tb.N, and Tb.Sp, highlighting its enhanced and more stable osteogenic performance compared to the as-received samples.

Histology in Fig. 7(d) likewise shows more continuous bone contact around the LIPSS–AgNPs surface, in agreement with the micro-CT trends. In addition, the double-fluorescence labeling shown in Figure 7e and quantified in Table 1 further supported active peri-implant bone formation and dynamic remodeling among the different groups. The apparently shallower implant in panel 7d results from a slightly oblique section rather than a difference in placement depth.

**Table 1:** Dual-color fluorescence analysis results.

	MAR	BFR/BS(um^2^/μm^2^/d)
As-received-AgNPs	0.8431	0.1454
Laser-Polished-AgNPs	0.8385	0.1832
LIPSS	0.9187	0.1913
LIPSS-AgNPs	0.8549	0.1691

Although surfaces with LIPSS structures alone outperformed as-received surfaces, the data exhibited greater variability. This could be attributed to the fact that LIPSS-modified surfaces without AgNPs experienced more pronounced inflammatory responses, which may have negatively impacted bone integration. Inflammation can interfere with the osteogenic process, leading to regional variations in bone formation [36]. Therefore, while LIPSS structures alone facilitate osteoblast activity, the incorporation of silver ions appears critical in mitigating inflammatory responses, thereby improving bone integration and overall healing.

Moreover, while conventional perspectives suggest that rough implant surfaces enhance bone integration via mechanical interlocking and cellular responses, the interplay between surface microtopography, inflammation, and osseointegration necessitates a careful balance. The LIPSS-AgNPs group, which effectively combines micro-topographical cues with antibacterial properties, emerges as the most promising strategy for optimal bone healing and long-term implant stability.

This dual-functional approach not only enhances osteogenic differentiation but also ensures sustained antibacterial efficacy, offering a robust solution for improving implant performance in clinical settings. The integration of hierarchical micro/nanostructures with controlled AgNPs release represents a significant advancement in implant surface engineering, addressing critical challenges in bone regeneration and infection control simultaneously.

## 4 Conclusion

In this study, we have developed a full-laser-enabled hierarchical surface engineering strategy that integrates laser precision polishing and femtosecond laser-induced periodic surface structures (LIPSS) followed by spatially controlled AgNPs deposition, to realize multifunctional synergy on 3D-printed titanium implants. This single-step fabrication strategy achieves defect-free surface finishing, antibacterial silver nanoparticle embedding, and osteoconductive microtopography, ensuring compliance with dental implant manufacturing standards. The LIPSS architecture enhanced fibroblast adhesion and alignment, while the hierarchical LIPSS configuration further promoted osteoblast differentiation and osseointegration. Simultaneously, the surface topography mechanically stretched bacterial membranes to facilitate Ag^+^ penetration, while the hierarchical structure modulated release kinetics to ensure prolonged antibacterial activity. Comprehensive in vitro and in vivo dog evaluations demonstrated that the above surface-modified titanium implants showed: (1) a 74.4% reduction in *Porphyromonas gingivalis* biofilm formation; (2) a 300% increase in effective Ag^+^ release duration for ensuring sustained antibacterial efficacy; (3)a 37.6% increase in gingival fibroblast proliferation; (4) enhanced osseointegration as evidenced by a 38.7% increase in bone-implant contact ratio. This innovative approach establishes a scalable and synergistically designed framework for next-generation bioactive implants, achieving unprecedented integration of osseointegration, infection resistance, and load-bearing capacity.

## CRediT authorship contribution statement

**Qirui Zhang:** Data curation, Formal analysis, Investigation, Validation, Writing – original draft. **Xinyue Zhang:** Investigation, Methodology, Resources, Validation. **Shanshan Liang:** Formal analysis, Investigation, Methodology, Resources, Validation. **Jiaru Zhang:** Data curation, Investigation, Methodology, Validation. **Qi Ma:** Investigation, Validation. **Yiyang Wang:** Data curation, Investigation, Methodology, Visualization. **Xing Li:** Investigation, Methodology, Resources, Validation. **Fusong Yuan:** Funding acquisition, Investigation, Resources, Visualization. **Yingchun Guan:** Conceptualization, Funding acquisition, Project administration, Supervision, Writing – review & editing. **Huaming Wang:** Funding acquisition, Resources, Supervision.

## Declaration of competing interest

The authors declare that they have no known competing financial interests or personal relationships that could have appeared to influence the work reported in this paper.

## Data Availability

The authors are unable or have chosen not to specify which data has been used.
