# Supplementary material for: Full-laser-enabled clean hierarchical structuring and multifunctional synergy for high-performance in vivo 3D-printed implants
Source: Mater Today Bio. 2026 Apr 3;38:103095. doi: 10.1016/j.mtbio.2026.103095 (PMC13090735; doi:10.1016/j.mtbio.2026.103095)
Supplement: Multimedia component 1 [file mmc1.doc]

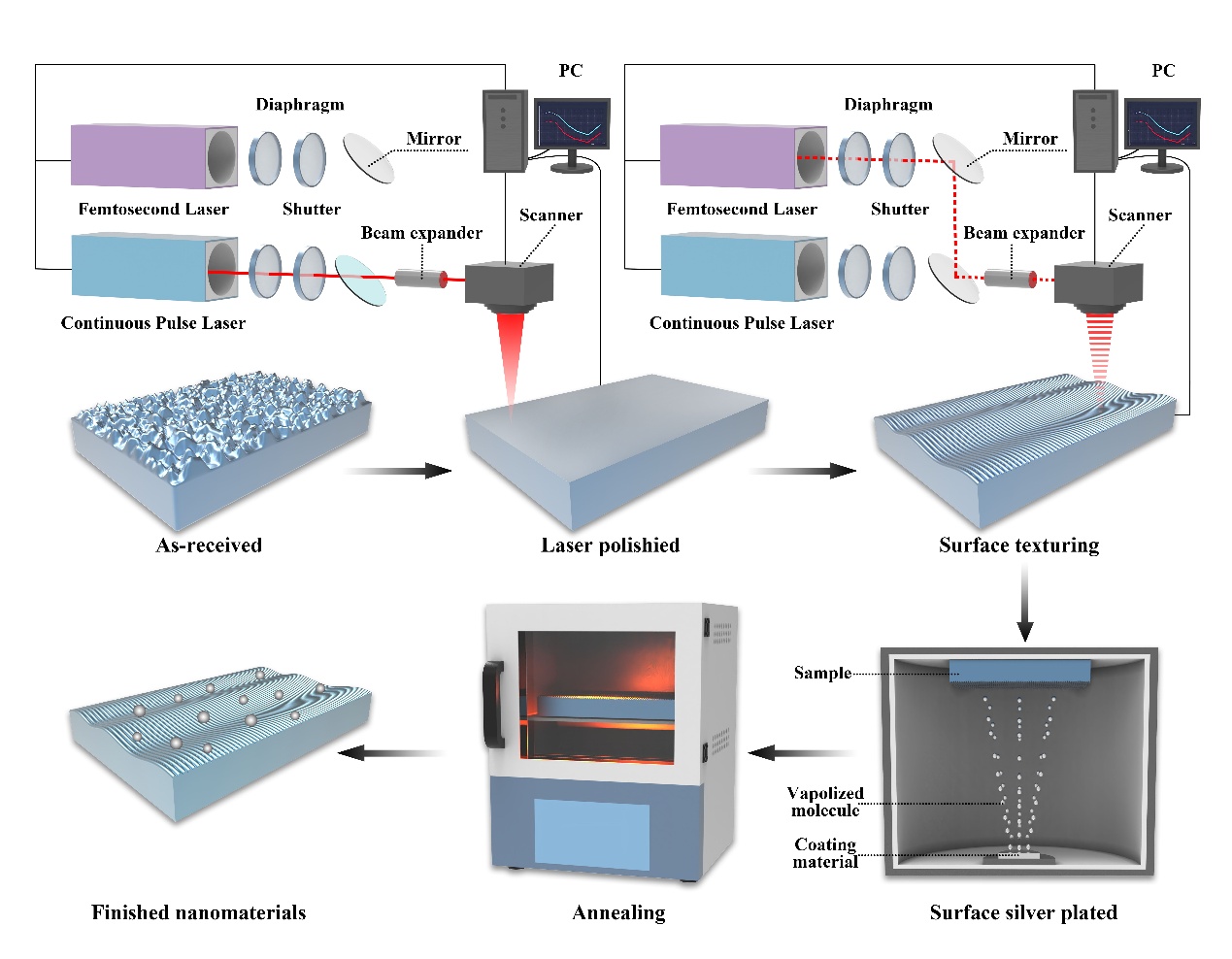


Figure S1. Schematic diagram of the implant preparation process.


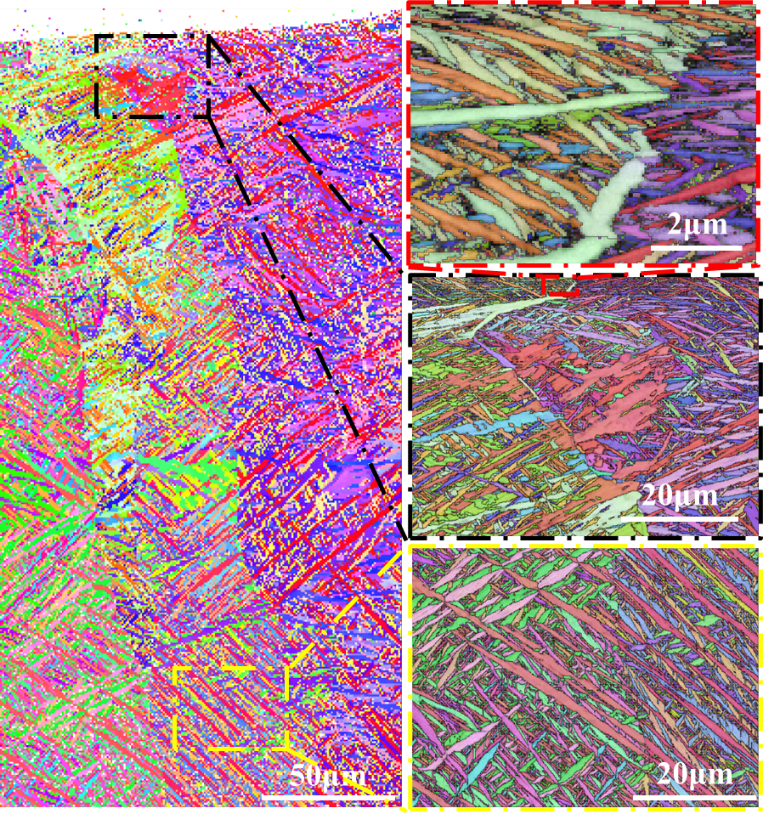


Figure S2. EBSD inverse pole figure (surface-normal coloring) maps of the near-surface region before and after laser polishing.


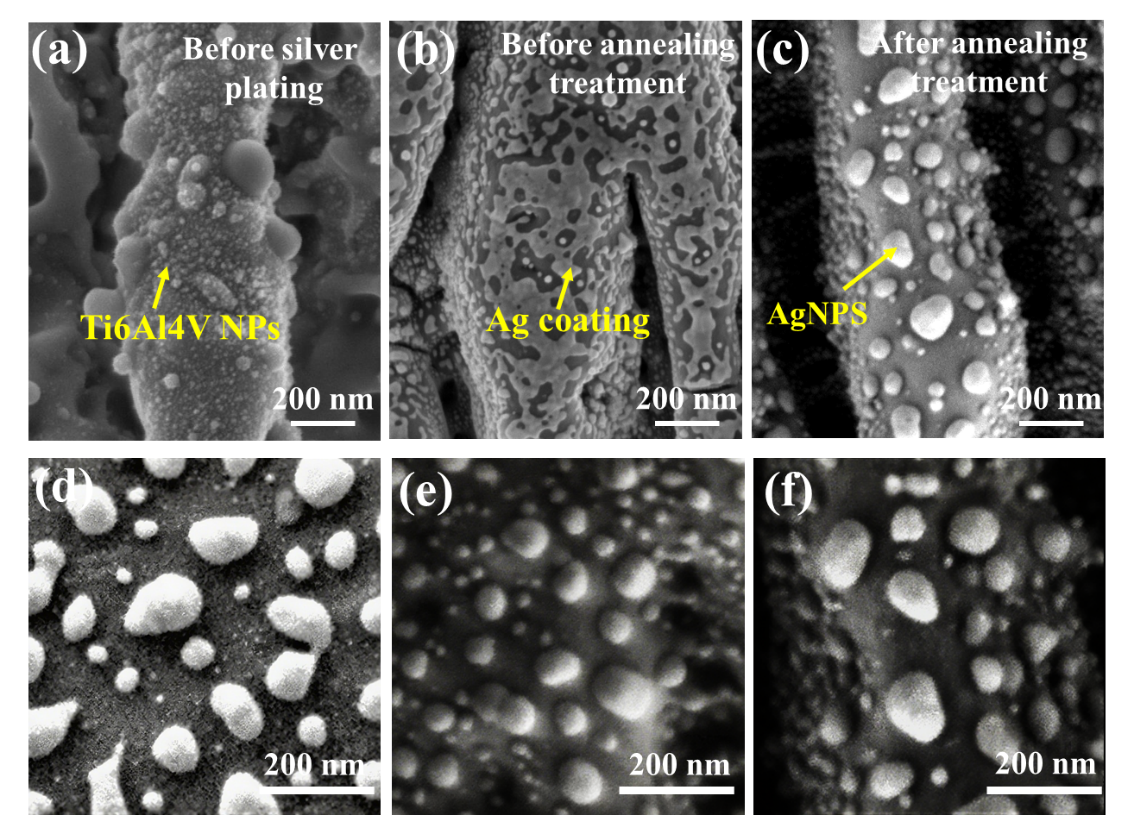


Figure S3. Surface characterization results of silver coatings under various treatment conditions:(a) Untreated surface before silver deposition,(b) Surface after silver deposition but before annealing,(c) Surface after silver deposition and annealing,(d) Surface after laser polishing with AgNPs,(e) Microcolumnar structure with AgNPs,(f) LIPSS structure with AgNPs.


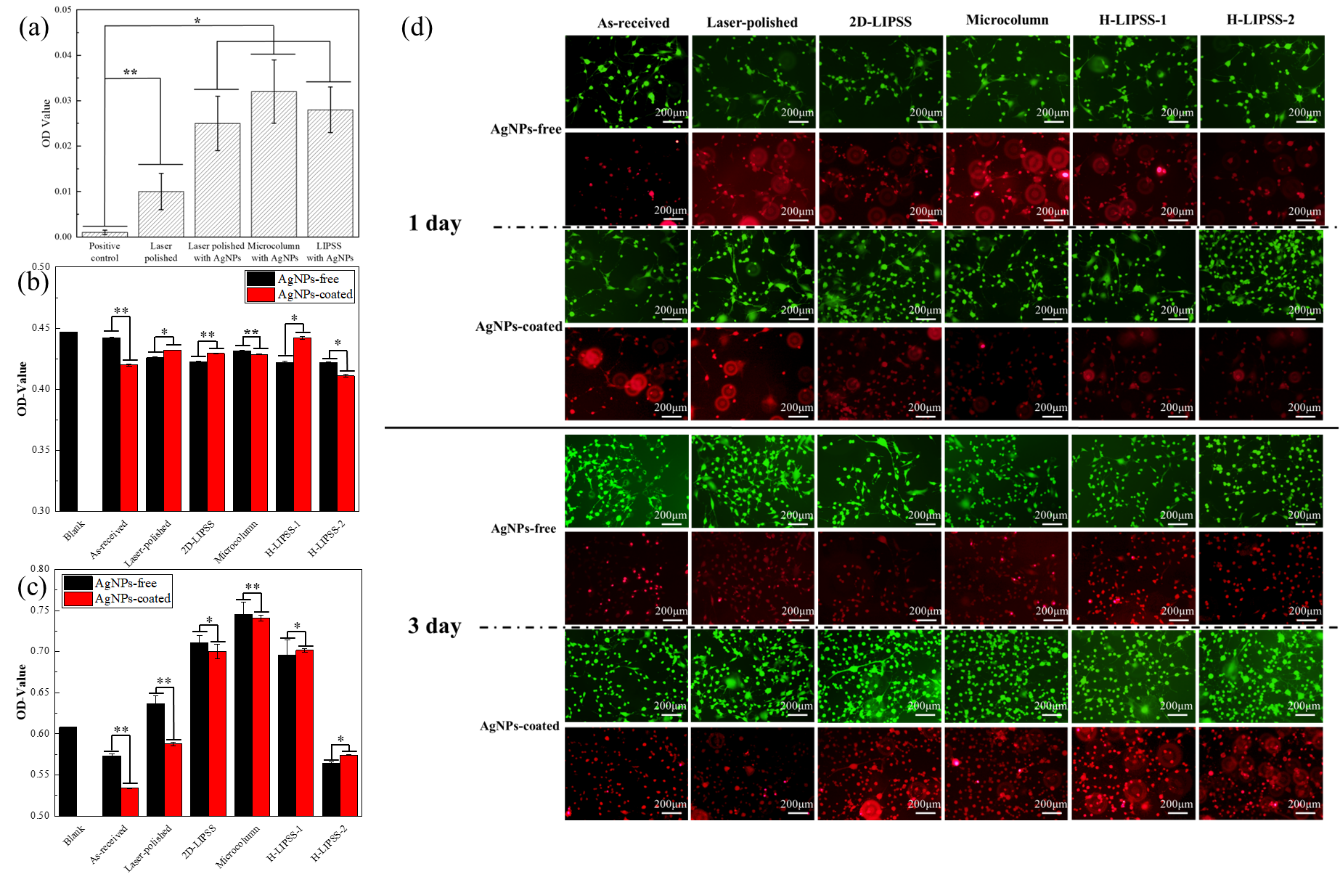


Figure S4. Biosafety assessment of AgNPs-free and AgNPs-coated samples by hemolysis assay and live/dead staining.(a) OD values at 540 nm of different samples for hemolysis rate evaluationError: Reference source not found；(b) Quantitative analysis of cell viability on different samples after 1 day of culture；(c) Quantitative analysis of cell viability on different samples after 3 days of culture；(d) Representative live/dead fluorescence images of cells cultured on different samples, showing live cells in green and dead cells in red. (*p-value < 0.05, **p-value < 0.01)


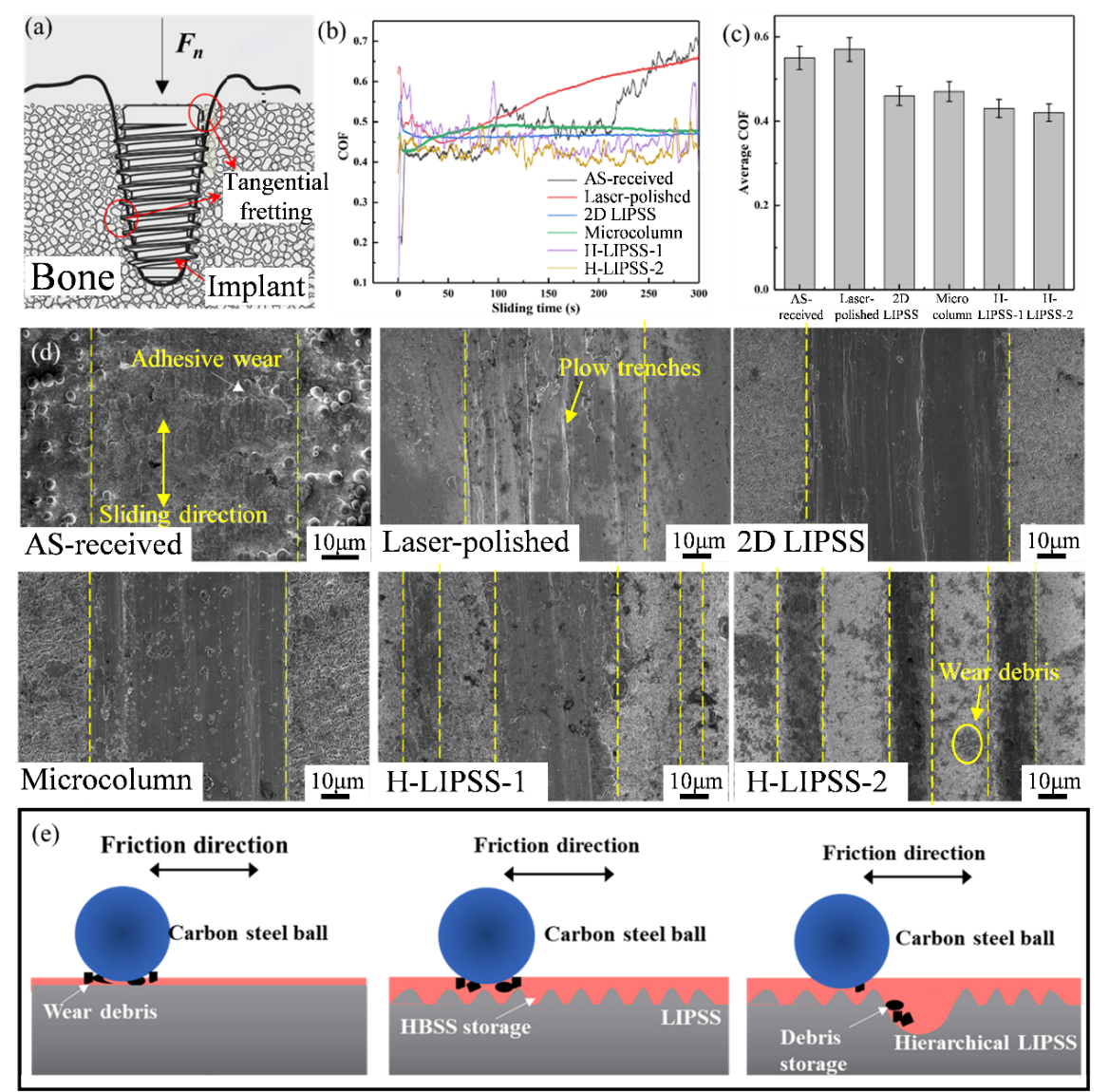


FigureS5. Friction and wear properties: (a) Force analysis on the dental implant/bone interface; (b) The COF of different samples under HBSS lubrication; (c) The wearing volume of different samples; (d) The wear morphology of different samples; (e) Wear mechanism analysis of the polishing surface, nanostructured surface and micro/nano structured surface.


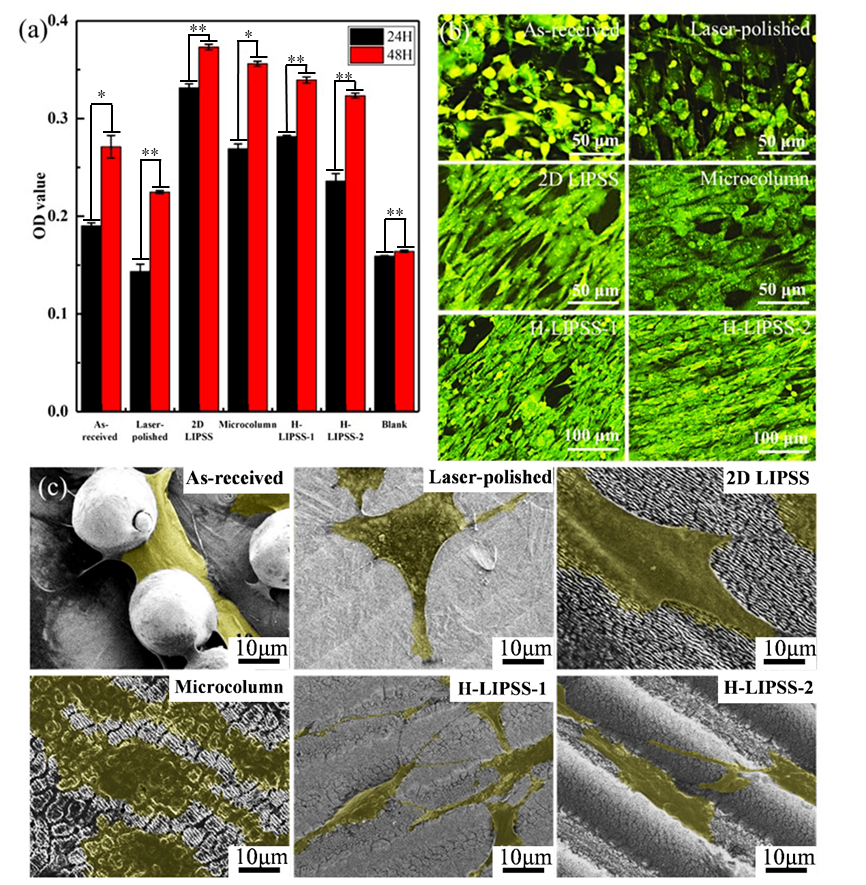


FigureS6. Effects of different surface morphology on fibroblasts: (a) The OD value of fibroblasts cultured for 24h and 48h; (b) Fluorescence images of fibroblasts cultured for 48h; (c) SEM images of fibroblasts cultured for 48h. (*p-value < 0.05, **p-value < 0.01)

**
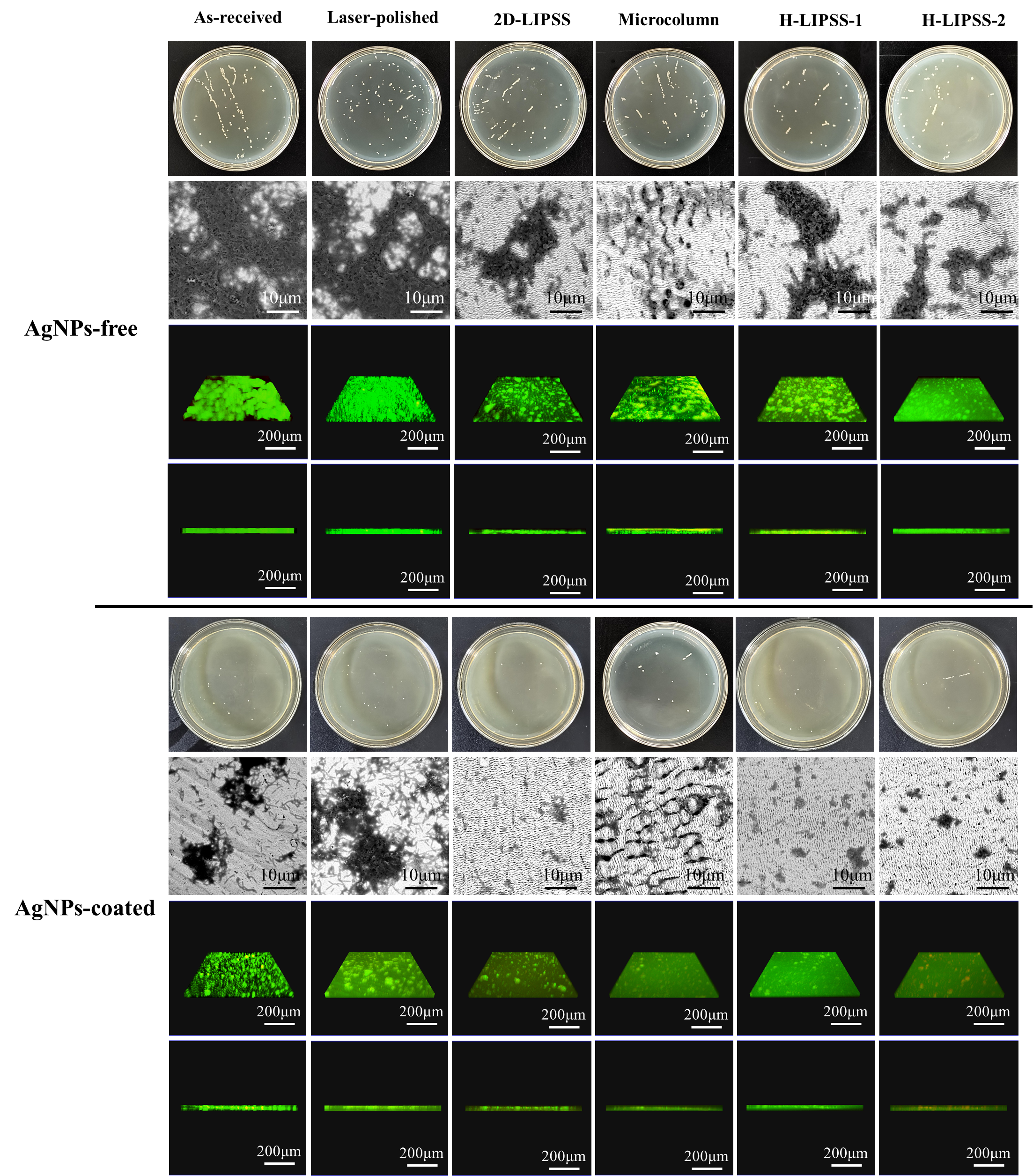
**

Figure S7. Representative agar plate images, SEM micrographs, and CLSM images of the multispecies oral biofilm formed on different sample surfaces after 48 h incubation under anaerobic conditions. The mixed bacterial model consisted of Streptococcus gordonii, Fusobacterium nucleatum, and Porphyromonas gingivalis. Agar plate images show the viable colonies recovered from each surface, while the corresponding SEM images reveal bacterial adhesion and biofilm morphology. CLSM images further provide visualization of the three-dimensional architecture of the formed biofilm, including reconstructed views and corresponding side-view images.


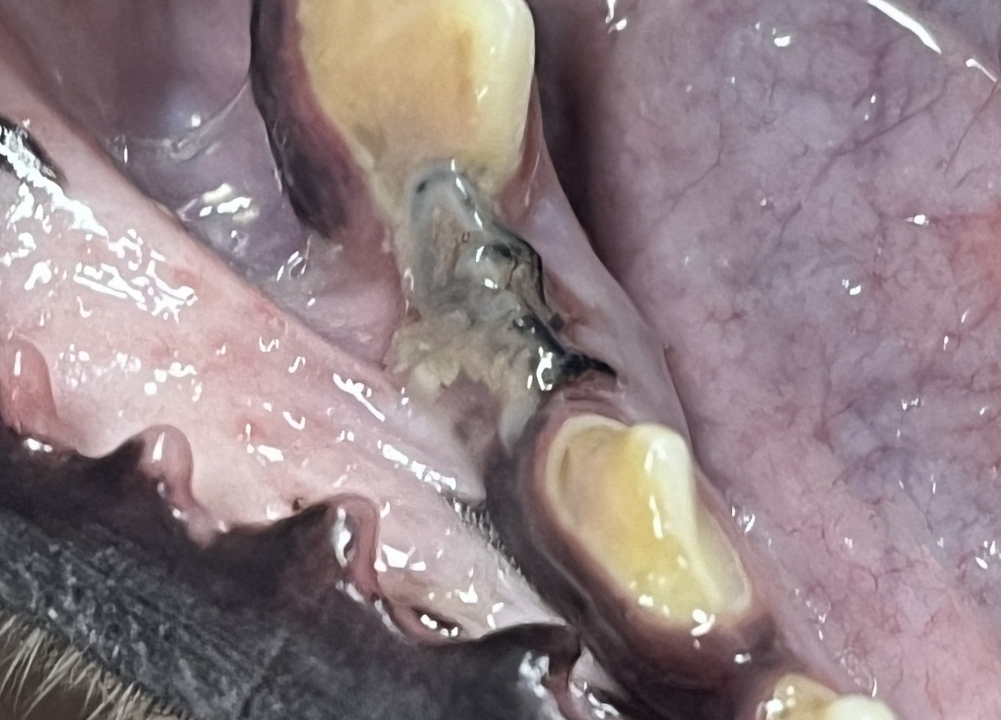


Figure S8. Intraoral photograph taken at week 1 in the ligature group, showing peri-implant edema, erythema and exudate at the implant neck. Image was acquired under anesthesia and is representative of four implant sites.

**Reference**

1. Lu L, Zhang J, Guan K, et al. Artificial neural network for cytocompatibility and antibacterial enhancement induced by femtosecond laser micro/nano structures. Journal of Nanobiotechnology, 2022, 20(1): 365. <https://doi.org/10.1186/s12951-022-01578-4>
